# TGF-β and BMPR2 Signaling in PAH: Two Black Sheep in One Family

**DOI:** 10.3390/ijms19092585

**Published:** 2018-08-31

**Authors:** Nina Rol, Konda Babu Kurakula, Chris Happé, Harm Jan Bogaard, Marie-José Goumans

**Affiliations:** 1 Department of Pulmonology, Amsterdam UMC, Vrije Universiteit Amsterdam, Amsterdam Cardiovascular Sciences, 1081HV Amsterdam, The Netherlands; n.rol@vumc.nl (N.R.); c.happe@vumc.nl (C.H.); hj.bogaard@vumc.nl (H.J.B.); 2 Department of Physiology, Amsterdam UMC, Vrije Universiteit Amsterdam, Amsterdam Cardiovascular Sciences, 1081HV Amsterdam, The Netherlands; 3 Department of Cell and Chemical Biology, Leiden University Medical Center, 2333ZA Leiden, The Netherlands; k.b.kurakula@lumc.nl

**Keywords:** pulmonary arterial hypertension, transforming growth factor β, bone morphogenetic protein, signaling, pathophysiology, treatment

## Abstract

Knowledge pertaining to the involvement of transforming growth factor β (TGF-β) and bone morphogenetic protein (BMP) signaling in pulmonary arterial hypertension (PAH) is continuously increasing. There is a growing understanding of the function of individual components involved in the pathway, but a clear synthesis of how these interact in PAH is currently lacking. Most of the focus has been on signaling downstream of BMPR2, but it is imperative to include the role of TGF-β signaling in PAH. This review gives a state of the art overview of disturbed signaling through the receptors of the TGF-β family with respect to vascular remodeling and cardiac effects as observed in PAH. Recent (pre)-clinical studies in which these two pathways were targeted will be discussed with an extended view on cardiovascular research fields outside of PAH, indicating novel future perspectives.

## 1. Introduction

Pulmonary arterial hypertension (PAH) is a condition defined by an increase in mean pulmonary artery pressure and characterized by remodeling of the pulmonary vasculature [1]. Abnormalities in vessel functionality and responses to stressors culminate in aberrant growth of endothelial cells (ECs) and smooth muscle cells (SMCs), leading to vascular obstruction and the formation of plexiform lesions. The increased pulmonary vascular resistance enhances the load upon the right ventricle (RV). The RV will compensate with hypertrophy, which progresses to RV-failure and death. Current available therapies for PAH mainly target vasoconstriction to reduce pressures and relieve the load, with some showing anti-proliferative effects in vitro. These drugs decelerate, but do not stop disease progression [2,3].

**Figure 1:**
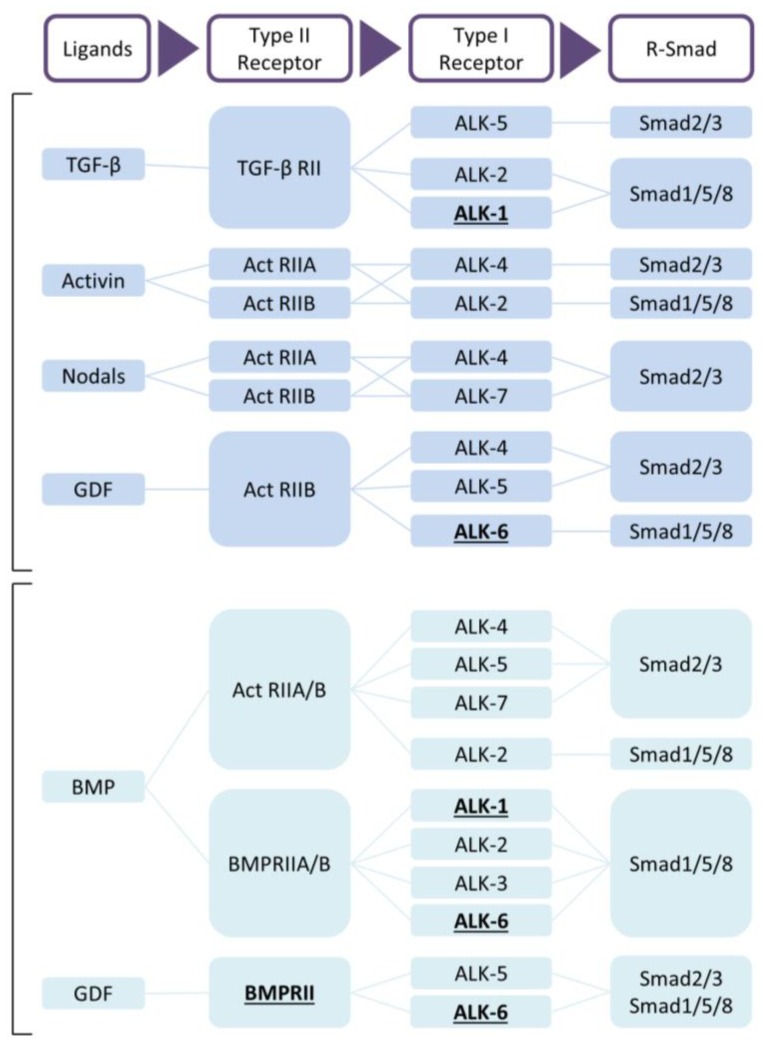
TGF-β and BMP signaling. Receptors with evidence of mutations in pulmonary arterial hypertension (PAH) are underlined [17]. Abbreviations: ActRII, activin receptor type II; ALK, activing receptor-like kinase; BMP, bone morphogenetic protein; GDF, growth/differentiation factor; TGF, transforming growth factor.

The transforming growth factor-β (TGF-β) family plays a major role in the initiation and progression of PAH. TGF-β is not only an important regulator of vascular remodelling and inflammation in the lung, but also of hypertrophy and fibrosis in the heart [4,5,6,7,8]. Of all receptors belonging to the TGF-β family (Figure 1), the bone morphogenetic protein type 2 receptor (BMPR2) is the most relevant for PAH. Mutations in the BMPR2 gene were the first discovered and most studied mutations underlying hereditary PAH to date [9,10]. BMPR2 is closely entangled with other members of the TGF-β family, but the roles of many of the ligands and receptors in the TGF-β family are still underappreciated in PAH. Although bone morphogenetic protein (BMP) ligands and their receptors play an important role in disease progression and could function as therapeutic targets [11], agents effectively decreasing TGF-β1 activity, together with selective TGF-β ligand traps open up new treatment possibilities [12,13,14,15,16].

Here, we give a comprehensive update on TGF-β signaling in PAH, summarized in Table 1. Furthermore, we provide insights into current (pre)-clinical studies targeting the TGF-β pathway in other diseases that may be useful in designing therapeutic strategies for the deadly condition of PAH.

## 2. TGF-β Signaling

Members of the TGF-β family are widely expressed in diverse tissues and play an essential role throughout life, starting from gastrulation and the onset of body axis asymmetry to organ-specific morphogenesis and adult tissue homeostasis [55,56,57,58]. At the cellular level, TGF-β family members regulate fundamental processes important for tissue homeostasis and embryogenesis, such as cell proliferation, differentiation, apoptosis, migration, adhesion, cytoskeletal organization, extracellular matrix production, in a context- and cell type-dependent manner. Consistent with this pleiotropic activity, disrupted TGF-β signaling is associated with several developmental disorders, cancer, auto-immune, cardiovascular and fibrotic diseases [55,56,57,59,60].

**Figure 2:**
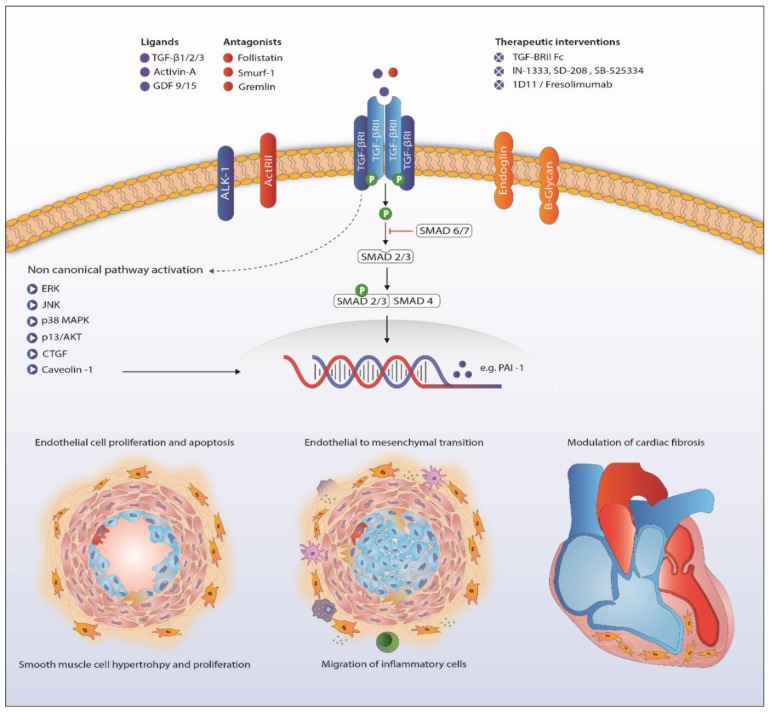
Proposed mechanism of TGF-β signaling in the pathogenesis of pulmonary arterial hypertension. Abbreviations: ActRII, Activin receptor type II; AKT, protein kinase B; ALK1, activin receptor-like kinase 1; CTGF, connective tissue growth factor; ERK, extracellular signal-regulated kinases; GDF, growth/differentiation factor; JNK, c-Jun N-terminal kinases; MAPK, mitogen-activated protein kinase; PAI-1, plasminogen activator inhibitor-1; TGF-β, transforming growth factor β. TGFBRII, TGF-β receptor type II.

The TGF-β family members are subdivided into two functional groups: the TGF-β group that comprises the three mammalian TGF-β isoforms, activins, nodals and some growth and differentiation factors (GDFs) and the BMP group that includes all BMPs and most GDFs (Figure 1) [57,59]. TGF-β family members form functional dimers, bind to heterotetrameric complex of type I and type II serine/threonine kinase transmembrane receptors and signal through both Smad-dependent and Smad-independent pathways [57,58,61,62] (Figure 2). In mammals, seven type I receptors, also known as activin receptor-like kinases (ALKs), and five type II receptors have been reported so far. To control duration and intensity of TGF-β signaling, agonists, antagonists, co-receptors and intracellular signaling play key roles in ligand access and posttranslational modification of the receptors and downstream mediators in a cell- and context-dependent manner [60,61,63]. TGF-β is secreted in its latent form and needs to be proteolytically processed before being able to bind to signaling receptors [4]. This complex activation mechanism could open up new therapeutic targets. TGF-β signals in most cells by binding to TβRII forming a complex with TβRI (or ALK5). Activins bind to activin receptor type IIA (ActRIIA) or ActRIIB in a complex with ALK4, while BMPs signal via BMP type II receptor (BMPRII), ActRIIA or ActRIIB, in combination with ALK1, 2, 3 or 6. Although TβRII/TβRI is the preferable high affinity signaling complex, in endothelial cells, TGF-β can also signal through TβRII/ALK1/ALK5 [64,65].

Upon complex formation, the activated type I receptor kinase will transduce the signal from the membrane to the nucleus by phosphorylating Smad transcription factors [61]. Smads are divided into three major classes: receptor-regulated Smads (R-Smads), common mediator Smad (co-Smad) and the inhibitory Smads (I-Smads). R-Smads (Smad1, Smad2, Smad3, Smad5 and Smad8) function as direct substrates for specific type I receptor kinases. ALK4, -5 and -7 phosphorylate Smad2 and Smad3, whereas Smad1, Smad5 and Smad8 become phosphorylated by the BMP type I receptors ALK1, -2, -3 and -6 [66]. Upon phosphorylation, R-Smads form a complex with the co-Smad, Smad4, and translocate to the nucleus. In the nucleus, Smad complexes engage in cooperative interactions with DNA and other DNA-binding proteins such as FAST1, FAST2, Fos/Jun and ATF2 to mediate the transcription of specific target genes [60,67]. The two I-Smads, Smad6 and Smad7, first identified in 1997 as vascular Smads, can compete with and inhibit R-Smads for type I interaction preventing phosphorylation [61,68]. Furthermore, they can induce proteasomal degradation of the type I receptor by recruiting Smurf1/2 E3 ubiquitin ligases [55,56,57,59,60]. For a more extensive description of TGF-β signaling, we refer to recent reviews [58,69,70,71,72,73,74,75,76,77].

## 3. Role of TGF-β Ligands in Pulmonary Arterial Hypertension

The presence of different TGF-β isoforms in the pulmonary vascular wall in the context of tissue remodeling in PAH was already described in 1994. Particularly, TGF-β3 is highly upregulated in both medial and intimal layers of remodeled pulmonary vessels [20]. More recent studies report that the increased presence of active TGF-β ligands co-localizes with SMCs in pulmonary arterioles and a strong expression of TGF-β1 in ECs and the interstitium of the plexiform lesions [46,78]. TGF-β signaling can directly inhibit BMP-Smad signaling in SMCs, and ligands from this side of the signaling balance can function as antagonists by competing for type II receptor binding [79,80]. Interestingly, pulmonary arterial ECs (PAECs) expressing a mutant BMPR2 release higher levels of TGF-β into the medium, thereby accelerating SMC growth [81]. As the quiescent effect that TGF-β typically has on SMC growth is impaired in PAH, the elevated TGF-β levels cause medial hypertrophy [82,83,84]. TGF-β-single nucleotide polymorphisms (SNP) on top of heterozygous BMPR2 mutation modulate the age of diagnosis and penetrance of familial PAH [45]. Other circulating ligands, such as activins and GDFs, are increased in PAH, as well, possibly stimulating cell growth and thereby contributing to pulmonary vascular remodeling [18,19,31,32,33,38]. The different animal models for pulmonary hypertension (PH) confirm the human pathology harboring more TGF-β and activins in the serum, pulmonary arteries and the RV in hypoxia or monocrotaline (MCT)-induced PH in rats [12,22,23,26]. The imperative role of TGF-β in PAH development is also illustrated by the dependency on this ligand in PAH associated with schistosomiasis in rats and required enhanced TGF-β signaling in a mouse model of scleroderma-related PH (SSc-PH) [24,85,86]. A recent study demonstrated that bone marrow-derived thrombospondin-1 causes *Schistosoma*- and hypoxia-induced pulmonary hypertension via activation of TGF-β [87].

## 4. Endothelial-to-Mesenchymal Transition in Pulmonary Arterial Hypertension

ECs can change their endothelial cobblestone morphology to a mesenchymal phenotype, a process referred to as endothelial-to-mesenchymal transition (EndoMT). In this process, ECs progressively lose their characteristics, i.e., cell-cell junctions and specific markers such as CD31, VE-cadherin and CD34 and gain markers such as α-SMA, collagen-I and vimentin migrate and invade into the surrounding tissues [88,89]. Although EndoMT takes place during embryogenesis where the transition contributes to the development of the valves of the heart, it does not occur under normal physiological circumstances [90]. An imbalance in the TGF-β/BMP axis and disturbed inflammation contribute to the induction of EndoMT [91]. EndoMT is stimulated by increased TGF-β receptor signaling and attenuated by intact BMPR2 signaling [21,92]. This process has been reported in pathologies such as inflammatory bowel disease, chronic kidney disease, cardiac fibrosis and portal hypertension [88,93,94,95]. In vitro, TGF-β-induced EndoMT in PAECs leads to higher migration rates, lower proliferation rates and decreased barrier integrity [91].

Both pre-clinical and clinical studies demonstrate that EndoMT plays a role in the pathogenesis of PAH [89,96]. EndoMT is also detected in the pulmonary vasculature of systemic sclerosis-associated PAH patients [91]. This study additionally shows that in vitro-induced EndoMT leads to reduced barrier integrity of PAECs with the production of pro-inflammatory cytokines such as IL-6, IL-8 and TNF-α and high trans-endothelial migration of immune cells.

In the pulmonary vasculature of MCT rats, overexpression of Twist-1 and VE-cadherin and repression of p120-catenin indicate the induction of EndoMT [21]. Rapamycin, an immunosuppressive drug, reverses experimental PH by inhibiting the migration of PAECs and reducing EndoMT markers. Ponatinib, a multi-target tyrosine-kinase inhibitor, attenuates TGF-β-induced EndoMT in human pulmonary microvascular ECs [96].

## 5. Receptors in Pulmonary Arterial Hypertension

Besides BMPR2 mutations, rare variants in other TGF-β receptor superfamily member genes are also associated with autosomal dominant familial PAH. Mutations in the type I receptor ALK1 and co-receptor endoglin are found in hereditary hemorrhagic telangiectasia (HHT)-associated PAH [35,36]. The increased prevalence of (h)PAH in HHT1 and HHT2 could be explained by the involvement of arteriovenous malformations, caused by ALK1 and ENG mutations, in the pathophysiology in both diseases [97,98,99]. Interestingly, in idiopathic PAH (iPAH) mRNA and protein levels of ALK1 and endoglin are specifically increased in ECs, leading to enhanced Smad1/5 phosphorylation (pSmad1/5) when stimulated with TGF-β, indicating a disturbed TGF-β/BMP balance [19]. In mice carrying a kinase-deficient TβRII in fibroblasts, the disturbed TGF-β signaling leads to pulmonary vasculopathy with medial thickening and mildly elevated pulmonary artery pressures [42]. TGF-β type III receptor (TβRIII) or β-glycan, a co-receptor acting as a reservoir of TGF-β2 for the type I and II receptors, is downregulated in familial PAH [28]. The functional consequences of these changes for the pathogenesis of PAH are yet unknown.

## 6. Canonical TGF-β Signaling in Pulmonary Arterial Hypertension

In the pulmonary vasculature, Smad2 phosphorylation after TGF-β receptor activation is increased, even though mRNA expression of Smad2 and Smad3 is decreased in whole lung lysates of PAH patients [27,39,45]. While pSmad2 (and not pSmad3) is likewise increased in the lungs of mice exposed to hypoxia, rats experimentally exposed to MCT develop PH 2–4 weeks after MCT injection, showing contrasting results with regards to canonical TGF-β signaling in the lung [25,31,46,100]. Some studies report increased pSmad2, while others show no change or even a decrease in Smad2 phosphorylation using Western blot analysis [12,37,43,46]. On the cellular, level increased Smad2 levels, driven by Activin A activation, are found in cultured SSc-PAH fibroblasts, responsible for collagen production [101]. Transgenic mice carrying an SMC specific dominant-negative BMPR2 gene do not show any alteration in Smad2 phosphorylation in lung tissue by Western blot analysis [102]. Although this could be due to technical differences between studies, it is possible that a BMPR2 mutation alone is not sufficient to regulate Smad2 phosphorylation.

Upon translocation into the nucleus, pSmad2 binds to the promoter of specific target genes like plasminogen activator inhibitor (PAI)-1, a well-acknowledged TGF-β target gene [103]. Interestingly, mRNA and protein expression of PAI-1 are decreased in iPAH, while circulating levels of PAI-1 are increased in both primary (idiopathic) and secondary PAH [49,50]. The latter is likely linked to the widespread development of thrombosis with intraluminal thrombin deposition [104]. The two widely-used experimental PH rat models, MCT and SuHx (VEGF receptor inhibitor Sugen combined with hypoxia), show conflicting results compared to the human situation, with increased mRNA expression of PAI-1 [12].

The co-Smad, Smad4, forms an intracellular complex with the TGF-β and BMP-mediated phosphorylated R-Smads and is needed for nuclear translocation [60]. Mutations in Smad4, together with ACVRL1 (ALK1) and ENG are causative of the vascular disorder HHT [105]. Nasim et al. report two independent iPAH cases with a missense and splice site mutation in Smad4, but no differential protein expression was found in PAECs and SMCs of iPAH patients [39,47]. In contrast with these human findings, Smad4 is reduced in MCT-induced PH on both the mRNA and protein level [43,48]. Transcription of the I-Smads, Smad6 and Smad7, is also reduced in lung tissue of these animals [48]. Differences in expression of I-Smads in human PAH tissue have not been reported to date. However, it has been shown that Smad6 is suppressed by the prostanoid Iloprost, thereby enhancing the intensity and duration of the TGF-β/Smad responses [106].

## 7. Non-Canonical TGF-β Signaling

Downstream signaling of TGF-β goes beyond phosphorylation of the Smad proteins. Activation of ERK, JNK/p38, Rho-like GTPases and PI3K/Akt is involved in the non-Smad pathway and also familiar in PAH research (Figure 2) [107,108,109,110]. Upregulation of these proteins in PAH has been shown before, although only a few in the context of disturbed TGF-β signaling [13,51,111]. Besides BMPR2 mutations, caveolin 1 (CAV1) mutations are a rare cause of PAH, influencing both canonical and non-canonical TGF-β/BMP signaling [36,52,112].

## 8. Downstream Targets of TGF-β in the Lung

Several reports demonstrated that vascular thrombosis plays an essential role in the pathophysiology of iPAH. Indeed, anticoagulation treatment confers a survival benefit in iPAH patients [113,114], perhaps because in situ thrombosis of pulmonary vessels may contribute to the pathogenesis of this disease [115,116]. Transcriptional activity of PAI-1 is elevated in patients with PAH along with other coagulation-associated genes. This increase in PAI-1 activity may explain impaired fibrinolysis in iPAH patients [116,117]. In contrast, two other studies demonstrated no change in PAI-1 activity in the serum of iPAH and chronic thrombo-embolic pulmonary hypertension (CTEPH) patients at rest or after venous occlusion [118,119]. However, the same group reported later that there is an increase in PAI-1 activity in female iPAH patients before and after venous occlusion [120]. The discrepancies between these studies may be caused by the use of different assays, gender differences and a low sample size per group. Given the heterogeneity in PAH patients, more studies are warranted to unravel the true function of PAI-1 in the pulmonary vasculature in PAH.

The inhibitor of DNA binding family of proteins (ID proteins) is a major downstream transcriptional target of BMP signaling [121]. In mammalian cells, four members of the Id family, Id1–4, have been identified so far. It has been reported that ID1, ID2 and ID3 are induced by BMPs in PAECs and SMCs through a canonical Smad-dependent pathway [11,106,121,122]. In adult organs, ID4 is mostly expressed in testis, brain and kidney and left out of the scope of this review [123]. Interestingly, BMP9 strongly induces the expression of ID proteins in PAECs, while BMP4 and BMP6 increase the expression of ID proteins in SMCs [121,124]. Mutations in BMPR2 strongly reduced the expression of ID proteins in both PAECs and SMCs. Unexpectedly, the expression of ID proteins along with BMPR2 expression are also reduced in PAECs and SMCs of some iPAH patients. In line with these observations in cultured cells, the expression of ID proteins is attenuated in experimental MCT-PH lungs, as well as in human lungs [48]. ID proteins also regulate the cell cycle and proliferation of SMCs in a BMP4-dependent manner [106,121]. Recently, BMP9 has been shown to selectively increase BMPR2, ID1 and ID3 proteins in endothelial cells in vitro and thereby decrease experimental PH in vivo, suggesting the potential involvement of these genes in PAH [11].

## 9. TGF-β Signaling in the Heart in Pulmonary Arterial Hypertension

Survival of PAH patients is determined by the ability of the RV to adapt to the increased pressures in the pulmonary vasculature [125]. The challenged RV suffers from neurohormonal activation, capillary loss inflammation, apoptosis, oxidative stress and metabolic shifts leading to hypertrophy and fibrosis [126]. Cardiac fibrosis is related to increased TGF-β signaling [127,128]. In rat, cardiac fibrosis induced by increased RV afterload, as seen in PH, is likely to be mediated through TGF-β-induced connective tissue growth factor signaling (Figure 2) [53,54]. The beneficial effects of carvedilol (β-blocker), iloprost (prostacyclin) and losartan (angiotensin receptor blocker) on RV function in animals are partly ascribed to attenuated TGF-β-mediated fibrosis [53,54,129]. Furthermore, nintedanib, a tyrosine kinase inhibitor known to inhibit TGF-β-mediated fibrosis, attenuated cardiac fibrosis in experimental pulmonary hypertension [130,131]

Hemnes et al. showed an upregulation of the TGF-β pathway in the RV of PAH patients by increased transcription of TGF-β3 [29]. TGF-β inhibition by either pan-TGFβ antibodies or specifically binding to TGF-β1 and TGF-β3 showed lowering of RV systolic pressures and attenuated RV hypertrophy in MCT and SuHx rat models [12,132]. One of the few studies investigating downstream TGF-β signaling in the heart in the context of PAH showed decreased phosphorylation of Smad2 in both RV and LV. This observation was independent of the presence of a BMPR2 mutation [40].

## 10. Therapeutic Interventions Relevant in PAH

In 13 preclinical and nearly twenty phase I–III clinical trials, TGF-β signaling is targeted to treat cancer and fibrotic diseases [72,77,133]. TGF-β signaling can be targeted mainly in three different ways in clinical trials: specific antibodies, antisense oligonucleotides and receptor kinase inhibitors. As ECs and SMCs in PAH produce excessive amounts of TGF-β, the use of these targets may decrease vascular remodeling through their inhibitory effect on these cells. In contrast to the clear role of TGF-β signaling in tumorigenesis, vascular diseases are more complex with simultaneous up- and down-regulation of the pathway and interactions with the BMP pathway [72].

Beneficial effects of inhibiting TGF-β ligands on pulmonary vascular and cardiac remodeling have previously been shown in experimental MCT- and hypoxia-induced rat PH models [12]. Targeting the ALK5 kinase with SD208, a drug known to suppress tumor metastasis in rodent models, ameliorated MCT-induced PH [134]. Beneficial anti-remodeling effects of prostacyclin analogues, used in PAH treatment strategies, can partly be explained by TGF-β inhibition [13,135]. How these effects in rat models can provide implications for the human disease are uncertain; besides increased availability of TGF-β ligands, different regulation patterns are observed. As TGF-β signaling is crucial for many physiological functions, prolonged inhibition of this signaling might lead to harmful side effects. Preclinical studies in PAH patient-derived cells could give valuable information about expected responses, illustrated by different effects in ECs and SMCs upon TGF-β stimulation [19,82,83].

## 11. Conclusions

In PAH, several mutations in components of the TGF-β/BMP signaling pathway have been identified. However, most research over the years has focused on BMP signaling, in particular BMPR2. Enhanced expression of TGF-β has been found systemically (i.e., in serum) and locally (i.e., in ECs and SMCs of the pulmonary vasculature) in PH patients and animal models. Furthermore, TGF-β has been shown to be involved in proliferation, inflammation, angiogenesis and fibrosis in lungs in PAH. In addition, TGF-β induces EndoMT, which is also involved in PAH, and as such, TGF-β could be interesting as a treatment target. Inhibition of TGF-β signaling, either directly or through targeting intermediates, may be a novel therapeutic strategy in PAH.

TGF-β signaling plays an essential role in vascular cells, immune cells and other cells such as epithelial cells in lungs. However, TGF-β signaling is very complex, as there are numerous ligands and diverse receptors that exhibit distinct functions in a cell- and context-dependent manner through interaction with other proteins, thereby affecting multiple signaling cascades. This intricate pathway is crucial for vessel wall homeostasis in many diseases, including PAH. Therefore, a deeper understanding of this pathway is necessary for the development of safer and efficient therapies for PAH.

In conclusion, overactive TGF-β signaling is an important regulator of pulmonary vascular remodelling in PAH, e.g., by balancing BMP signaling. TGF-β inhibitors have entered clinical trials for treatment of cancer and fibrotic diseases with encouraging first clinical results. The future of specific TGF-β inhibitors are promising and open new challenges in PAH research.

## Figures and Tables

**Table 1:** TGF-β signaling in pulmonary arterial hypertension in human tissue and animal models.

		Serum		Lung Tissue/Vessel		Heart Tissue		EC		SMC		References
**Ligands**												
TGF-β1	*mRNA*	=		=	↑ ^a,b,d^		↑ ^b^	=		↑		[12,18,19,20,21,22,23,24,25,26]
	*Protein*	↑		↑	↑ ^a,b,d^		↑ ^b^					
TGF-β2	*mRNA*				↓ ^b^							[12,20]
	*Protein*			=								
TGF-β3	*mRNA*			↓		↑						[12,20,27,28,29,30]
	*Protein*			↑	= ^b,c^ ↑ ^a^							
Activin A	*mRNA*											[31]
	*Protein*	↑	↑ ^a^									
GDF 9/15	*mRNA*			↑								[32,33,34]
	*Protein*	↑		↑								
**Type I receptors**												
ALK1	*mRNA*			↑				↑		=		[19,35,36,37]
	*Protein*			↑	↑↓ ^b^			↑		=		
ALK5	*mRNA*			=↑		=		=		=		[19,21,38,39,40,41]
	*Protein*			=				=				
**Type II receptors**												
TGFBRII	*mRNA*				↓ ^b^↑ ^a,c^							[25,42,43,44]
	*Protein*				↓ ^b^			=		=		
ActRII	*mRNA*											[31]
	*Protein*				↑ ^a^							
**Co-receptors**												
β-glycan	*mRNA*	=		↓								[28,38]
	*Protein*											
Endoglin	*mRNA*			=	↓ ^b^			↑		=		[19,43]
	*Protein*			↑				↑		=		
**Canonical signaling**												
Smad2	*mRNA*			↓								[12,27,31,37,39,40,41,43,44,45,46]
	*Protein*				↑↓= ^a,b^	↓		↑				
Smad3	*mRNA*			↓	↓ ^b^							[12,13,27,31,41,43]
	*Protein*				↑ ^a,b^↓ ^b^							
Smad4	*mRNA*			↓	↓ ^b^							[27,37,39,43,47,48]
	*Protein*				↓ ^b^			=		=		
Smad6/7	*mRNA*				↑ ^a^↓ ^b^							[37,44,48]
	*Protein*				↑ ^a^							
PAI-1	*mRNA*	↑		↓	↑ ^b^							[12,13,49,50]
	*Protein*			↓	↑ ^b^							
**Non-canonical signaling**												
MAPKs	*mRNA*							↑				[51]
	*Protein*											
Cav1	*mRNA*											[36,37,52]
	*Protein*			↓	= ^b^							
CTGF	*mRNA*				↓ ^b^		↑ ^c,e^					[43,53,54]
	*Protein*				↓ ^b^		↑ ^c,e^					

Grey boxes indicate findings in tissue of pulmonary arterial hypertension patients; white boxes indicate findings in experimental animal models. ↑, increase in comparison to controls; ↓, decreased in comparison to controls; =, no change in comparison to controls. (**^a^**) Hypoxia-induced PH in rat, (**^b^**) monocrotaline-induced PH in rat, (**^c^**) Sugen hypoxia-induced PH in rat, (**^d^**) *Schistosoma*-induced PH in mice, (**^e^**) pulmonary artery banding in rats. Increases in Smad protein regard phosphorylation. Abbreviations: ActRII, Activin receptor type II; ALK1, activin receptor-like kinase 1; ALK5, activin receptor-like kinase 5; Cav1, caveolin-1; CTGF, connective tissue growth factor; EC, endothelial cell; GDF, growth/differentiation factor; MAPKs, mitogen-activated protein kinase; PAI-1, plasminogen activator inhibitor-1; SMC, smooth muscle cell; TGF-β, transforming growth factor β; TGFBRII, TGF-β receptor type II.
